# XYZ color data on the visual degradation of vegetables

**DOI:** 10.1016/j.dib.2019.105079

**Published:** 2020-01-03

**Authors:** Carlos Arce-Lopera, Katsunori Okajima, Yuji Wada

**Affiliations:** a Universidad Icesi, Cali, Colombia; b Yokohama National University, Yokohama, Japan; c Ritsumeikan University, Kyoto, Japan

**Keywords:** Vegetable, Visual degradation, Optical measurement, XYZ color space, Material perception

## Abstract

This data article includes the visual stimuli used to model the freshness perception of four different vegetable textures, namely a cabbage, a carrot, a strawberry and a spinach. All four vegetables were optically measured during their degradation process in a humidity, temperature and light controlled environment. The visual data is in csv format for convenient usage. Each data point represents a pixel value using the hardware independent XYZ color space. The total size of the data can be related to an equivalent image of 1360 × 1024 resolution. Additionally, using the calibration data of an LCD-Display, the respective RGB color space images were derived from the XYZ data as an example. For interpretation and discussion, please see the original article entitled “Model of vegetable freshness perception using luminance cues” [1].

**Table:** Specifications Table

Subject	Experimental and Cognitive Psychology
Specific subject area	Consumer Perception
Type of data	Image (.bmp format) .CSV Files
How data were acquired	Data was recorded using a 2D luminance and chromaticity analyzer (TOPCON UA1000)
Data format	Raw
Parameters for data collection	Images were taken in a light, humidity and temperature controlled environment.
Description of data collection	Four types of vegetable (cabbage, strawberry, carrot and spinach) were measured during three days of degradation.
Data source location	City: Yokohama Country: Japan
Data accessibility	With the article
Related research article	Arce-Lopera C.A., Masuda T., Kimura A., Wada Y., Okajima K. Model of vegetable freshness perception using luminance cues. Food Quality and Preference. 2015; 40B: 279–286.

**Table:** 

**Value of the Data** • The data shared in this data article is the raw XYZ values of the degradation process of four vegetables in a controlled environment. These data are particularly useful to enable faithful luminance and color reproduction in psychophysical experimentation. Previous initiatives to facilitate standardization and comparability across studies using food images limit their contribution to sharing images in hardware-dependent color spaces such as the RGB color space [2]. XYZ color information is hardware-independent allowing that the images can be reproduced exactly as the real objects in the measuring scene. • These data are beneficial to researchers in different fields, such as in food science, in consumer perception, in human and animal visual perception and in computer and information science. • Furthermore, this data can be used in the development of further experiments to test different types of consumer perceptions. The visual stimuli can be exploited in the development of a multitude of experimental settings in sensory science research. For example, an experiment could test subjects' perceived quality assumptions using eye tracking software. Further psychophysical experiments could explain the influence of visual cues on taste or nutrient estimates and the relationship with visual texture degradation. • Additionally, the visual stimuli could be used to describe the relationship between product visual characteristics and color simulations of different types of vegetable textures. The data collected from the 2D luminance and chromaticity analyzer is unique and no other research group has shared publicly this type of data with the scientific community.

## 1 Data description

The dataset in this article contains image raw data of the degradation process of four different vegetables obtained from a 2D Luminance and Chromaticity Analyzer (TOPCON UA-1000). The data is in comma-separated value (.csv) format to ensure ease of use. The file naming protocol adopted was simply to concatenate the type of vegetable with the number of hours from the start of the measuring session. For example, the image raw data of the cabbage vegetable at 8 h of degradation can be found in the cabbage8h.csv file (see Table 1). For each vegetable, the corresponding raw data was organized in a.zip file for compression and organization purposes. Therefore, four different.zip files, one for each vegetable, is shared with this data article (see XYZCabbage.zip, XYZCarrot.zip, XYZStrawberry.zip and XYZspinach.zip). Additionally, as examples of the possible visual stimuli, bitmap images were calculated for all the.csv files using the calibration data of an LCD-monitor, an Eizo ColorEdge CG245W 24-Inch LCD Monitor (see RGB_ExampleData.zip). The.csv files which represent the raw XYZ color data are organized as follows:

• The X color channel data is from the row 2 to 1025.
• The Y color channel data is from the row 1028 to 2051.
• The Z color channel data is from the row 2054 to 3077.

**Table 1:** List of shared files with description.

Filename	Type of vegetable	Time of degradation	Type of data
cabbage0h.csv	Cabbage Leaf	0 hours	XYZ values
cabbage1h.csv	Cabbage Leaf	1 hour	XYZ values
cabbage3h.csv	Cabbage Leaf	3 hours	XYZ values
cabbage5h.csv	Cabbage Leaf	5 hours	XYZ values
cabbage8h.csv	Cabbage Leaf	8 hours	XYZ values
carrot0h.csv	Carrot	0 hours	XYZ values
carrot3h.csv	Carrot	3 hours	XYZ values
carrot6h.csv	Carrot	6 hours	XYZ values
carrot12h.csv	Carrot	12 hours	XYZ values
carrot18h.csv	Carrot	18 hours	XYZ values
spinach0h.csv	Spinach	0 hours	XYZ values
spinach3h.csv	Spinach	3 hours	XYZ values
spinach15h.csv	Spinach	15 hours	XYZ values
spinach38h.csv	Spinach	38 hours	XYZ values
spinach66h.csv	Spinach	66 hours	XYZ values
strawberry0h.csv	Strawberry	0 hours	XYZ values
strawberry12h.csv	Strawberry	12 hours	XYZ values
strawberry24h.csv	Strawberry	24 hours	XYZ values
strawberry48h.csv	Strawberry	48 hours	XYZ values
strawberry72h.csv	Strawberry	72 hours	XYZ values

## 2 Experimental design, materials, and methods

The selection of the types of vegetable was guided by two main aspects. First, their consumption popularity in raw form in Japan and second, their degradation in visual appearance with time. Therefore, the four selected vegetables were: the cabbage, the carrot, the spinach and the strawberry. The vegetables were randomly selected from a local market and the day of purchase was the initial day of the measuring session. The vegetables were measured without any cleaning or preparation, just careful handling and unpacking was performed. All vegetables were ready to consume in their raw forms. The optical measurement equipment was set in a 30-degree Celsius temperature-controlled and 6% humidity-controlled environment. Two leaves of a white cabbage (*Brassica oleracea* var. capitata), a cut carrot (Daucus carota subsp. sativus), one leaf of spinach (Brassica rapa var. perviridis) and 6 different strawberries (Fragaria x ananassa Duch.) were measured using the Topcon UA1000, a luminance and chromaticity analyzer. The measurement equipment took data each 30 minutes for several days for each setting. Then, five samples for each vegetable were selected, including a high and a low freshness reference. The high freshness reference was chosen to be the first measurement at 0 hours. For each vegetable, the low freshness reference was different and chosen arbitrarily to be the time when the vegetable did not seem appetizing anymore. For the cabbage, the low freshness reference was set to 8 hours after the start of the measuring session. For the carrot, the spinach and the strawberry, it was set to 18, 66 and 72 hours, respectively. For more details on the stimuli, refer to Ref. [1].

**Fig. 1:**
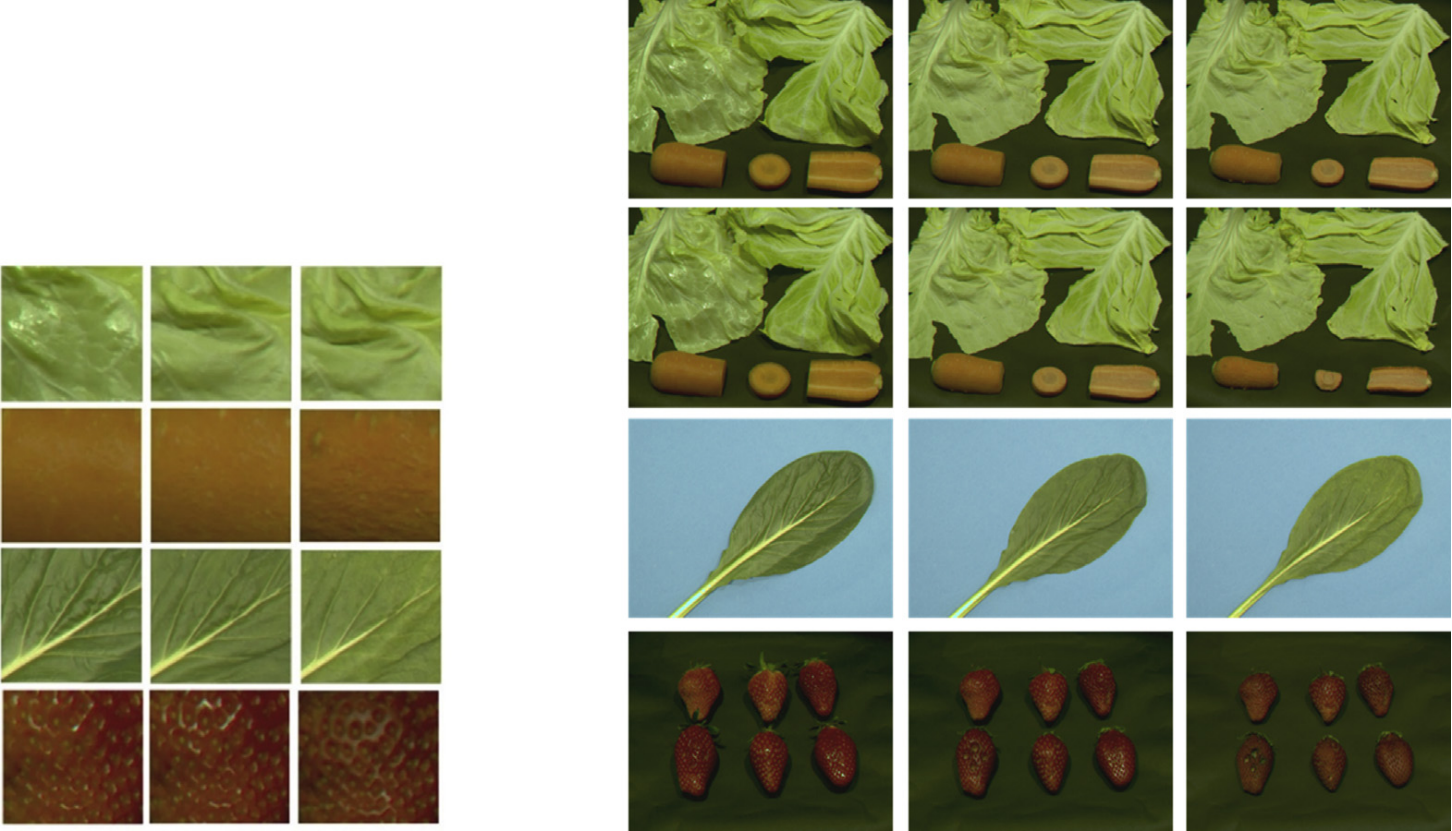
Data of the four food textures at different degradation time. On the left side are the visual food textures used in Ref. [1]. On the right side, there are some images representing the shared data.

As an example, the resulting data was processed using the calibration data of an LCD monitor to derive the corresponding RGB values for each data point (see RGB_ExampleData.zip). Here, the shared visual data is not a cropped version of the original images [1] but the full raw results of the optical measurements. This way future researchers can use shape deformation information as well as visual texture in their experiments. Fig. 1 shows examples of the shared data in comparison with the stimuli used in Ref. [1]. For the cabbage and carrot texture, the measurement data was obtained in the same session. For this reason, in Fig. 1, the two cabbage leaves and the three portions of the carrot are in the same image.

Generally, the scientific community struggles with visual stimuli acquired with optical cameras due to the difficulty to calibrate the camera sensors [3]. Indeed, the camera calibration is needed to be able to relate the real optical measures of an object in a particular scene with the camera output representation (commonly, in RGB color space). Additionally, commercially available cameras modify the sensor raw outputs in a variety of ways which increases the difficulty to reverse engineer what the camera manufacturer does inside the camera. Therefore, the camera calibration process is a costly but necessary procedure [4]. To be able to surpass this problem, more specialized and expensive optical measurement tools have been created, such as the Topcon UA1000, a 2D luminance and chromaticity analyzer. The output of this measuring tool is a calibrated 2D image in the hardware independent XYZ color space. Therefore, after a color conversion to a particular RGB color space, which depends on the display device, the images can be reproduced faithfully in that calibrated monitor.

## Conflict of Interest

The authors declare that they have no known competing financial interests or personal relationships that could have appeared to influence the work reported in this paper.
